# Supplementary material for: Baseline Serum Biomarkers Predict Response to a Weight Loss Intervention in Older Adults with Obesity: A Pilot Study
Source: Metabolites. 2023 Jul 17;13(7):853. doi: 10.3390/metabo13070853 (PMC10385260; doi:10.3390/metabo13070853)
Supplement: Supplementary file 1 [file metabolites-13-00853-s001.zip › Supplementary Materials Checklist.pdf]

Checklist of Supplementary Material:

Supplementary tables

The supplementary tables are provided as Excel spreadsheets.

1. Supplementary Table S1. UPLC-MS Signals that differentiated responders from non-responders that were identified or annotated using the in-house physical standards library or annotated using public databases.
2. Supplementary Table S2. Candidate pathways, enriched in Functional Analysis module in Metaboanalyst 5.0 using the Mummichog Algorithm, that differentiated responders from non-responders.

Supplementary figures

3. Supplementary Figure S1. Scores plot of PCA of UPLC-MS metabolomics data.

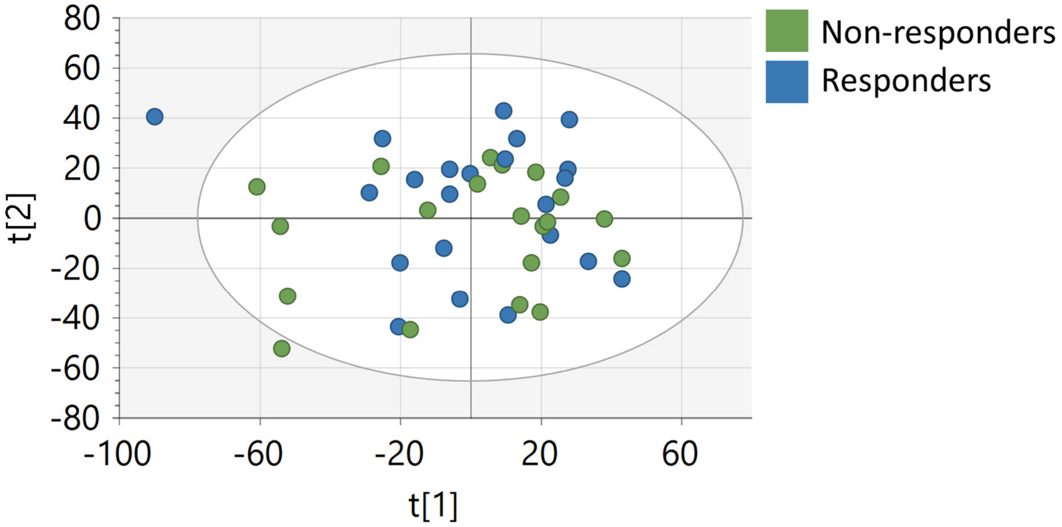

Figure S1. PCA of baseline samples of responders and non-responders.
